# Beyond Lesion Control: A Narrative Review of Targeted Therapy and Surgical Decision-Making in Children With Vascular Anomalies Across Function, Growth, and Perioperative Care

**DOI:** 10.7759/cureus.110532

**Published:** 2026-06-09

**Authors:** Omer Altamimi, Ibrahim Altamimi, Rahaf Alhowidi, Ghaida M Alosaimi, Eman M Alzahrani, Aseel Ashri, Yara H Essa, Yara F Aljefri, Sultan Khoja, Lamar A Alsaigh, Abdulkreem Al-Juhani

**Affiliations:** 1 Department of Surgery, Faculty of Medicine, King Abdulaziz University Hospital (KAUH), Jeddah, SAU; 2 Department of Surgery, Aldireyah Hospital, Riyadh, SAU; 3 College of Medicine, Almaarefa University, Riyadh, SAU; 4 College of Medicine, Umm Al-Qura University, Makkah, SAU; 5 Department of Plastic and Reconstructive Surgery, College of Medicine, King Abdulaziz University, Jeddah, SAU; 6 Department of Vascular Surgery, King Abdulaziz University Hospital (KAUH), Jeddah, SAU; 7 Department of Medicine and Surgery, Batterjee Medical College, Jeddah, SAU; 8 Department of Forensic Medicine, Forensic Medicine Center, Jeddah, SAU; 9 Department of Surgery, College of Medicine, King Abdulaziz University, Jeddah, SAU

**Keywords:** debulking, pediatric, reconstructive surgery, sirolimus, vascular anomalies

## Abstract

Pediatric vascular anomalies are heterogeneous disorders that may cause pain, bleeding, infection, deformity, airway compromise, impaired mobility, psychosocial distress, and long-term functional morbidity. Surgery has traditionally played a central role in selected lesions through excision, debulking, staged reconstruction, and functional restoration. However, in children, operative decision-making is complicated by infiltrative anatomy, bleeding risk, lymphatic leakage, recurrence, scarring, growth potential, and the possibility of repeated procedures across childhood. The emergence of targeted therapies, particularly sirolimus for complex slow-flow malformations and alpelisib for PIK3CA-related overgrowth spectrum and selected vascular malformations, has shifted the management paradigm. These agents should not be viewed only as medical alternatives to surgery; rather, they may reshape surgical timing, extent, risk, and goals. This narrative review examines how targeted therapy may influence reconstructive surgical decision-making in children with vascular anomalies. We propose a child-centered model in which surgery may be performed, facilitated, delayed, avoided, or modified after targeted therapy. We also introduce the PERFORM framework to guide multidisciplinary assessment of patient age, lesion extent, treatment response, functional threat, operative risk, reconstruction required, and long-term follow-up. Future studies should move beyond radiologic response and report pediatric outcomes that matter: function, growth, quality of life, recurrence, revision surgery, and family burden.

## Introduction and background

Vascular anomalies comprise a heterogeneous group of disorders historically separated into vascular tumors and vascular malformations, a biologic distinction first formalized by Mulliken and Glowacki and later refined through the International Society for the Study of Vascular Anomalies (ISSVA) classification systems [1-3]. In children, correct classification is not merely semantic; it determines imaging interpretation, genetic testing, natural history, procedural risk, and therapeutic strategy. Radiologic-pathologic correlation has therefore become central to modern pediatric vascular anomaly care, particularly when lesions cross anatomical compartments or mimic tumors, inflammatory disease, or congenital overgrowth syndromes [4].

The pediatric burden of vascular anomalies extends beyond visible deformity. Complex lesions may cause pain, bleeding, recurrent infection, ulceration, airway compromise, feeding difficulty, impaired mobility, coagulopathy, disfigurement, and psychosocial distress. Health-related quality of life studies have shown that children with congenital vascular malformations may experience substantial physical and emotional morbidity, while broader systematic evidence confirms that vascular malformations can significantly affect patient-reported outcomes [5,6]. The Outcome Measures for Vascular Malformations initiative has emphasized the need for standardized outcome domains and condition-specific patient-reported measures, reflecting a shift from clinician-centered lesion assessment toward child- and family-centered outcomes [7,8].

Historically, management has relied on observation, compression, laser therapy, sclerotherapy, embolization, and surgery, either alone or in combination. Over the last decade, however, targeted therapy has changed the therapeutic landscape. Sirolimus, an mTOR inhibitor, has demonstrated clinical benefit in complicated vascular anomalies, especially slow-flow, lymphatic, venous, and combined malformations, across early case series, prospective trials, and pediatric cohorts [9-12]. More recently, molecular discoveries in the PI3K/AKT/mTOR pathway have supported precision treatment for selected vascular malformations and overgrowth syndromes; alpelisib, a PI3Kα inhibitor, has shown activity in PIK3CA-related overgrowth spectrum and severe vascular anomalies [13,14].

Despite this progress, the reconstructive implications of targeted therapy remain underdeveloped in the literature. Most studies emphasize symptom improvement, radiologic response, or drug safety, while fewer address whether targeted therapy changes the surgical pathway. Early surgical reports suggest that sirolimus may be used as an adjunct in selected slow-flow malformations, and perioperative pediatric data have specifically evaluated wound complications in children receiving sirolimus around surgical procedures [15,16]. This narrative review, therefore, reframes targeted therapy as a surgical decision-modifying tool in pediatric vascular anomalies. The aim is to examine how therapy may influence surgical timing, debulking, reconstruction, functional outcomes, growth considerations, and perioperative care.

## Review

Methods

A structured narrative literature review was conducted to synthesize evidence relevant to targeted therapy and reconstructive surgical decision-making in pediatric vascular anomalies. PubMed/MEDLINE, Scopus, Web of Science, and Google Scholar were searched using combinations of the following terms: “vascular anomalies,” “vascular malformations,” “lymphatic malformation,” “venous malformation,” “arteriovenous malformation,” “PIK3CA-related overgrowth spectrum,” “children,” “pediatric,” “sirolimus,” “alpelisib,” “trametinib,” “targeted therapy,” “surgery,” “reconstruction,” “debulking,” “resection,” “perioperative,” and “wound healing.”

Priority was given to pediatric clinical trials, cohort studies, case series, case reports, multidisciplinary reviews, and surgical studies that discussed targeted therapy, surgical timing, perioperative outcomes, functional reconstruction, or long-term pediatric considerations. Adult studies were considered only when they addressed concepts directly relevant to pediatric practice, such as outcome measurement, molecular mechanism, or perioperative decision-making. Reference lists of key articles were manually screened to identify additional relevant sources. A narrative review design was selected because the objective was not to estimate pooled treatment effects, but to provide a clinically oriented synthesis and propose a practical framework for multidisciplinary pediatric decision-making.

Review

Classification, Burden, and the Child-Centered Surgical Problem

The first step in surgical planning is accurate diagnosis. Pediatric vascular anomalies differ widely in flow dynamics, tissue involvement, natural history, complication profile, and response to therapy. Imaging plays a critical role in distinguishing venous, lymphatic, arteriovenous, and combined malformations, as well as in defining the relationship of the lesion to airway, orbit, genitourinary structures, neurovascular bundles, muscle, bone, and growth centers [17]. This matters surgically because the same visible deformity may represent a compressible venous malformation, a microcystic lymphatic malformation, a high-flow arteriovenous malformation, or a syndromic overgrowth disorder.

Venous malformations may also be complicated by localized intravascular coagulopathy, which has direct implications for bleeding risk, thrombosis, pain, and perioperative planning [18]. Complex cases are therefore best managed in multidisciplinary vascular anomaly teams that integrate pediatrics, plastic and reconstructive surgery, pediatric surgery, dermatology, hematology-oncology, interventional radiology, radiology, genetics, anesthesiology, rehabilitation, psychology, and nursing [19]. However, vascular anomaly care remains variable across centers, and national survey data suggest that team composition, referral patterns, and resource availability are not uniform [20]. Recent pediatric imaging reviews similarly emphasize that clinical-radiologic integration is essential before choosing medical, interventional, or surgical treatment [21].

Conventional Surgery and Why Complete Excision Is Not Always the Pediatric Endpoint

Surgery remains essential in selected pediatric vascular anomalies. Indications include airway obstruction, visual compromise, recurrent bleeding, pain, ulceration, infection, functional impairment, localized mass effect, genital or perineal morbidity, severe deformity, and residual tissue after medical or interventional control. Depending on subtype and site, surgery may involve excision, debulking, staged reconstruction, flap coverage, skin grafting, scar revision, contour restoration, or function-preserving reconstruction.

However, vascular anomalies are often infiltrative rather than encapsulated. In lymphatic malformations, management varies across observation, sclerotherapy, systemic therapy, surgery, or combined approaches, and systematic evidence confirms significant heterogeneity in pediatric treatment strategies [22]. Retrospective pediatric surgical-interventional series have shown that lesion morphology, location, and extent influence whether surgery, sclerotherapy, combined therapy, or observation is selected [23]. Earlier treatment reviews also recognized that head and neck lymphatic malformations, mixed lesions, and lesions involving critical structures are particularly challenging, with recurrence and repeated procedures remaining important concerns [24].

For children, the surgical objective is not always complete eradication. A child-centered surgical endpoint may be safer: preservation of airway, feeding, speech, mobility, continence, cosmesis, limb function, school participation, psychosocial wellbeing, and future growth. This distinction is important because aggressive excision may create morbidity that exceeds the benefit of lesion reduction, especially in diffuse or growth-sensitive anatomical regions.

Molecular Rationale for Targeted Therapy

The modern era of vascular anomaly care has been shaped by molecular discovery. Early genetic work established that vascular malformations are often biologically driven rather than purely structural developmental accidents [25]. Somatic activating mutations in PIK3CA have been identified in lymphatic and other vascular malformation-overgrowth disorders, connecting phenotype to the PI3K/AKT/mTOR pathway [26]. Additional work showed that somatic PIK3CA mutations may cause venous malformations, while experimental and human studies supported the role of PIK3CA activation in sporadic venous malformations [27,28].

Recent advances in molecular biology have further strengthened the concept that many vascular malformations are genetically driven disorders characterized by somatic mosaic mutations involving interconnected signaling pathways [26]. In addition to PIK3CA alterations, abnormalities involving TEK/TIE2, AKT, and RAS/MAPK signaling have been increasingly implicated in venous, lymphatic, and overgrowth-associated vascular anomalies [25]. These discoveries have accelerated interest in precision medicine approaches, where molecular profiling may eventually influence not only drug selection but also reconstructive timing, operative planning, and long-term surveillance strategies in children with diffuse or anatomically complex disease [27]. As targeted therapies continue to evolve, future pediatric management may increasingly shift from anatomy-based treatment alone toward biologically informed multidisciplinary decision-making [28].

This molecular framework creates a new surgical question. If a lesion is pathway-driven, then targeted therapy may reduce inflammatory activity, pain, leakage, swelling, bleeding tendency, or overgrowth before a surgeon enters the field. Conversely, if therapy produces symptom control without meaningful anatomical resolution, surgery may remain necessary for residual functional obstruction or deformity. Therefore, targeted therapy should be understood not only as a drug response but also as a modifier of surgical indication.

Sirolimus and the Shift From Procedural Rescue to Medical-Surgical Planning

Sirolimus has the strongest pediatric evidence base among targeted therapies for complex vascular anomalies. Early pediatric series described clinical improvement in difficult lesions that were previously refractory to conventional approaches [9]. Subsequent prospective work demonstrated efficacy and safety signals in complicated vascular anomalies, while later multicenter and randomized clinical evidence supported sirolimus as a treatment option for selected slow-flow malformations in children [10-12]. Retrospective pediatric series have further highlighted both indications and limitations, emphasizing that response varies by subtype, extent, and clinical target [29,30].

The surgical implication is nuanced. Sirolimus may reduce pain, swelling, lymphatic leakage, infection frequency, or functional compromise, potentially allowing surgery to be postponed, narrowed, or avoided. Yet, sirolimus may also be used before planned surgery to reduce lesion activity or after surgery to control residual disease. The literature does not support a simplistic “medical therapy versus surgery” model. Instead, sirolimus creates a continuum in which the surgical team must repeatedly reassess whether debulking, resection, reconstruction, or surveillance best serves the child’s function and long-term development.

Despite the expanding use of sirolimus in pediatric vascular anomalies, treatment response remains variable across lesion subtypes and anatomical locations [22]. While many children experience improvement in pain, swelling, lymphatic leakage, and recurrent inflammatory symptoms, complete radiologic resolution is uncommon in extensive or infiltrative lesions [18-20]. Furthermore, some studies have reported recurrence or clinical progression following dose reduction or discontinuation, suggesting that sirolimus may function primarily as long-term disease control rather than definitive cure in selected patients [18].

Long-term pediatric monitoring, therefore, remains essential during therapy. Recent studies have highlighted concerns regarding infectious complications, pharmacokinetic variability in younger children, and the need for individualized monitoring protocols during prolonged treatment courses [20-26]. Additional preventive strategies, including infection prophylaxis in selected immunosuppressed patients receiving PI3K/AKT/mTOR pathway inhibitors, have also been proposed in recent literature [29]. These considerations are particularly important in children requiring repeated procedures or extended multidisciplinary care throughout growth and development.

Alpelisib and Precision Treatment in Overgrowth-Associated Vascular Anomalies

Alpelisib has expanded the concept of targeted therapy beyond symptom suppression toward genotype-informed disease modification. In infants with severe PIK3CA-related overgrowth spectrum, alpelisib has been reported to produce clinical, biological, and radiologic improvement, raising the possibility of earlier disease stabilization in life-threatening or anatomically extensive conditions [31]. Prospective cohort evidence in severe vascular malformations harboring PIK3CA and TEK mutations has also suggested meaningful clinical benefit with limited toxicity in selected patients [32]. More recent data indicate that PI3Kα inhibition may have activity across vascular anomalies with diverse genotypes and phenotypes, although long-term pediatric surgical implications remain incompletely defined [33].

Perioperative management of targeted therapies remains insufficiently standardized across pediatric vascular anomaly centers [32]. Although sirolimus may reduce inflammatory activity and lesion burden before surgery, concerns persist regarding wound healing, immunomodulation, infection risk, and postoperative tissue recovery [30]. Consequently, perioperative practices vary considerably between institutions, with some multidisciplinary teams temporarily interrupting therapy before major reconstructive procedures, while others continue treatment in selected high-risk lesions requiring ongoing disease control.

Additional perioperative challenges include infectious monitoring, nutritional optimization, metabolic surveillance, and individualized drug-level assessment during prolonged therapy [30-33]. Recent pediatric studies have also emphasized the importance of multidisciplinary perioperative planning involving surgeons, anesthesiologists, hematologists, dermatologists, pharmacists, and interventional radiologists when managing children with diffuse vascular malformations or medically complex overgrowth syndromes [34-36]. However, evidence-based perioperative protocols remain limited, highlighting an important area for future collaborative research.

For reconstructive surgeons, alpelisib is particularly relevant in overgrowth-associated disease. Children with PIK3CA-related overgrowth spectrum may require repeated debulking, orthopedic procedures, soft tissue contouring, limb or trunk reconstruction, and operations for functional impairment. If targeted therapy reduces disease activity or overgrowth burden, it may change the timing and extent of these procedures. However, the surgical literature has not yet standardized how to report whether alpelisib reduces operative frequency, changes flap or graft requirements, improves symmetry, or delays revision during growth [36].

MEK Inhibition and High-Flow Disease: Emerging Implications

High-flow arteriovenous malformations remain among the most difficult lesions to treat surgically because incomplete treatment may worsen progression, and extensive resection may carry major bleeding and functional risk. Genotype-guided treatment of a pediatric arteriovenous malformation with trametinib has illustrated the potential role of pathway-directed therapy in selected cases [34]. Mosaic RAS/MAPK variants have also been linked to sporadic vascular malformations that may respond to targeted therapy, further supporting the biological rationale for MEK inhibition in selected lesions [35]. Pediatric reports using imaging and clinical monitoring to assess genotype-targeted therapy response suggest that the field is moving toward lesion-specific, mutation-informed management [36].

At present, MEK inhibition should be viewed as an emerging strategy rather than an established reconstructive adjunct. Recent reviews of targeted medical therapies emphasize that molecular diagnosis is increasingly shaping treatment selection, dosing, monitoring, and future trial design across vascular anomalies [37]. The reconstructive question is whether these therapies can stabilize high-flow disease enough to reduce operative risk, convert an unresectable lesion into a staged surgical candidate, or avoid mutilating procedures during childhood. The targeted therapies most relevant to pediatric vascular anomaly care, their molecular targets, pediatric indications, reconstructive implications, and perioperative concerns are summarized in Table 1.

**Table 1:** Targeted therapies relevant to pediatric vascular anomalies and their reconstructive implications

Therapy	Main target/pathway	Representative pediatric relevance	Potential surgical implication	Key pediatric/perioperative concerns
Sirolimus	mTOR inhibition within the PI3K/AKT/mTOR pathway	Complex slow-flow, lymphatic, venous, and combined malformations; used in complicated pediatric vascular anomalies [9-12,29,30]	May reduce symptoms, swelling, leakage, infection frequency, or lesion activity; may facilitate, delay, modify, or occasionally avoid debulking/reconstruction [15,16,29,30]	Infection monitoring, mucositis, cytopenias, dyslipidemia, drug-level monitoring, and uncertain perioperative interruption practices [10-12,16]
Alpelisib	PI3Kα inhibition	PIK3CA-related overgrowth spectrum and selected PIK3CA/TEK-driven vascular malformations [13,14,31-33]	May modify timing and extent of debulking, contouring, and staged reconstruction in overgrowth-associated lesions [14,31-33]	Hyperglycemia, rash, mucosal toxicity, access, long-term pediatric safety, and limited surgical outcome data [14,31-33]
MEK inhibitors, e.g., trametinib	RAS/MAPK pathway inhibition	Selected genotype-driven arteriovenous malformations and RAS/MAPK-related vascular malformations [34-37]	Potential future role in stabilizing high-flow disease before high-risk surgery; evidence remains emerging [34-37]	Limited pediatric reconstructive evidence, toxicity monitoring, and unclear perioperative protocols [34-37]
Genotype-directed targeted therapy more broadly	Lesion-specific pathway inhibition	Increasingly relevant as somatic mutations are identified in vascular malformations [25-28,37]	May shift planning from anatomy-only surgery to biologically informed medical-surgical sequencing [25-28,37]	Requires access to genetics, molecular interpretation, longitudinal monitoring, and multidisciplinary decision-making [19,20,37]

How Targeted Therapy Modifies Surgery: Performed, Facilitated, Delayed, Avoided, or Modified

The central novelty of this review is the proposal that targeted therapy changes the surgical pathway in five clinically distinct ways. First, surgery may still be performed despite targeted therapy. This occurs when the child has persistent airway obstruction, visual compromise, feeding difficulty, pain, recurrent bleeding, localized mass effect, severe deformity, or residual tissue requiring reconstruction. In this scenario, targeted therapy is supportive but not definitive. Second, surgery may be facilitated by targeted therapy. Preoperative treatment may reduce swelling, leakage, inflammatory activity, pain, or lesion burden, allowing a safer or more limited operation. This principle is suggested by reports using adjuvant sirolimus in the surgical management of slow-flow malformations and by pediatric perioperative data addressing sirolimus exposure around surgical procedures [15,16]. Third, surgery may be delayed. In children, delay can be beneficial when early surgery risks growth disturbance, scarring, neurovascular injury, or repeated revision. A medically stabilized lesion may allow the team to wait until the child is older, anatomy is larger, anesthesia risk is lower, or reconstructive options are more favorable. Fourth, surgery may be avoided. Avoidance should not be equated with cure. Rather, it means that medical response has changed the risk-benefit balance such that immediate debulking or resection is no longer justified. This may be appropriate when pain, bleeding, infection, leakage, or functional symptoms improve sufficiently. Fifth, surgery may be modified. Instead of complete excision, the surgical plan may shift toward focused functional debulking, staged reconstruction, contour correction, limited excision of symptomatic components, or combined interventional radiology and reconstructive procedures. This is especially relevant in diffuse, syndromic, or growth-sensitive lesions. For surgical planning, targeted therapy may alter the pediatric operative pathway in five practical ways: surgery may be performed, facilitated, delayed, avoided, or modified, as summarized in Table 2.

**Table 2:** Five ways targeted therapy may modify surgery in children with vascular anomalies

Surgical pathway	Meaning	Pediatric example	Child-centered surgical implication	Supporting evidence signal
Surgery performed	Operation remains necessary despite targeted therapy	Persistent airway, visual, feeding, genital, mobility, bleeding, pain, or deformity-related indication	Surgery is justified by functional threat or residual reconstructive need rather than lesion size alone	Surgical and interventional management remains central in selected pediatric lesions [22-24]
Surgery facilitated	Therapy improves surgical conditions before operation	Reduced lymphatic leakage, swelling, or inflammatory activity before debulking	May allow safer, more limited, or staged reconstruction	Adjuvant sirolimus has been reported in surgical slow-flow malformations; perioperative sirolimus data address wound outcomes [15,16]
Surgery delayed	Therapy stabilizes the disease until a better pediatric time point	Young child with diffuse lesion near growth-sensitive anatomy	May reduce early scarring, repeated anesthesia, growth disturbance, or premature revision	Pediatric cohorts show targeted therapy can provide symptom control in complex lesions [9-12,29,30]
Surgery avoided	Medical response changes the risk-benefit balance against immediate operation	Pain, leakage, bleeding, or infection improves sufficiently with drug therapy	Avoidance means surveillance and reassessment, not cure	Sirolimus and alpelisib studies report clinical improvement in selected complex anomalies [10-14,31-33]
Surgery modified	Surgical goal changes after response	Extensive excision becomes focused functional debulking or staged contour reconstruction	Aligns surgery with function, growth, and long-term morbidity reduction	Molecularly targeted therapy may reshape medical-surgical sequencing [25-28,37]

Pediatric Outcomes Beyond Lesion Control

Radiologic response alone is an incomplete endpoint in children. A lesion may shrink without improving function, or symptoms may improve without major volumetric change. Pediatric outcomes should therefore include airway, feeding, speech, vision, mobility, pain, recurrent infection, bleeding, limb function, facial symmetry, genital or perineal function, school participation, psychosocial wellbeing, family burden, and need for future surgery.

The OVAMA work has already moved the field toward standardized symptom, appearance, and quality-of-life measurement [7,8]. Responsiveness testing of condition-specific vascular malformation outcome measures further supports the need for instruments that can detect clinically meaningful change over time [38]. Future pediatric surgical studies should report not only whether a drug reduces lesion size but also whether it changes the child’s surgical trajectory: fewer operations, safer debulking, reduced wound complications, improved function, delayed revision, or better long-term growth. Future medical-surgical studies should therefore report standardized pediatric outcomes across lesion control, surgical morbidity, function, growth, patient-reported outcomes, and long-term trajectory, as summarized in Table 3.

**Table 3:** Pediatric outcomes that should be reported in future medical-surgical studies

Outcome domain	Suggested outcomes	Why it matters in children	Supporting evidence
Lesion control	Size, radiologic response, flow characteristics, progression, recurrence	Radiologic change helps define treatment response but is insufficient alone	Imaging-based classification and response assessment are central to care [4,17,21]
Surgical outcomes	Blood loss, operative time, wound complications, lymphatic leakage, infection, flap/graft need, revision surgery	These outcomes determine whether targeted therapy truly changes operative morbidity	Perioperative and surgical pediatric studies remain limited but directly relevant [15,16,22-24]
Functional outcomes	Airway, feeding, speech, vision, continence, mobility, pain, bleeding, infection frequency	Function often matters more than complete lesion removal in children	Pediatric vascular malformations affect physical and emotional functioning [5,6]
Growth outcomes	Facial symmetry, limb length, soft tissue overgrowth, skeletal distortion, timing of revision	Children may require repeated reassessment as anatomy grows	Overgrowth-related lesions are biologically linked to PI3K pathway disease [13,14,26,31]
Patient-reported outcomes	Symptoms, appearance, quality of life, psychosocial wellbeing, family burden	Child and family experience may differ from clinician-rated lesion response	OVAMA and related work emphasize standardized patient-centered outcomes [7,8,38]
Long-term trajectory	Number of procedures, delayed surgery, avoided surgery, transition to adult care, durability after drug interruption	A therapy is surgically meaningful if it changes the lifetime burden of operations	Longitudinal reporting remains a major gap across targeted therapy studies [29,30,37,38]

Perioperative Care and Multidisciplinary Coordination

Targeted therapy introduces new perioperative questions. For sirolimus, concerns include immunomodulation, mucositis, cytopenias, dyslipidemia, infection risk, and theoretical wound-healing effects. Pediatric surgical data have not shown a clear association between sirolimus and wound complications in one cohort, but evidence remains limited and should not be overgeneralized [16]. For alpelisib, perioperative issues may include hyperglycemia, rash, mucosal toxicity, nutritional status, infection monitoring, and drug access. For MEK inhibitors, the pediatric reconstructive evidence is even more limited, and potential adverse effects require subspecialty monitoring.

No universal rule currently defines when to stop or restart targeted therapy around surgery. Decisions should be individualized according to lesion type, drug indication, surgical magnitude, infection risk, wound complexity, nutritional status, hematologic parameters, metabolic profile, and urgency of operation. Multidisciplinary planning is especially important when surgery involves the airway, genitourinary structures, major vessels, extensive soft tissue, or anticipated staged reconstruction.

Proposed PERFORM Framework

To translate the literature into practical clinical reasoning, we propose the PERFORM framework for surgical decision-making in children with vascular anomalies receiving targeted therapy: Patient age and growth potential, Extent and anatomical site of anomaly, Response to targeted therapy, Functional threat, Operative risk, Reconstruction required, and Multidisciplinary follow-up.

The framework is not intended to replace expert vascular anomaly team judgment. Rather, it offers a structured way to document why surgery is performed, facilitated, delayed, avoided, or modified. It also encourages outcome reporting that reflects pediatric priorities, including growth, function, quality of life, and future reconstructive burden. The proposed PERFORM framework provides a structured method for documenting how targeted therapy informs pediatric surgical decision-making, as outlined in Table 4.

**Table 4:** PERFORM framework for targeted therapy-informed surgical decision-making ISSVA: International Society for the Study of Vascular Anomalies

PERFORM component	Clinical question	Surgical decision it informs	Documentation recommended
Patient age and growth potential	Is the child in a growth-sensitive phase where surgery may affect symmetry, function, or future reconstruction?	Whether to operate now, delay, or stage reconstruction	Age, pubertal stage when relevant, growth asymmetry, anticipated revision burden
Extent and anatomical site	Is the lesion localized, diffuse, infiltrative, airway-threatening, limb-threatening, genital, craniofacial, or functionally disabling?	Extent of excision, debulking, flap/graft need, and procedural risk	ISSVA subtype, flow status, MRI/ultrasound findings, relation to critical structures [2-4,17,21]
Response to targeted therapy	Has therapy improved symptoms, size, bleeding, leakage, pain, infection, or function?	Whether surgery should be performed, facilitated, delayed, avoided, or modified	Drug, dose, duration, adverse effects, clinical response, imaging response [9-14,29-33]
Functional threat	Is there airway, feeding, speech, vision, mobility, continence, pain, bleeding, or infection risk?	Whether surgery is urgent, elective, staged, or avoidable	Functional assessment, symptom scores, school/activity limitation, family report [5-8,38]
Operative risk	What is the risk of bleeding, coagulopathy, lymphatic leak, wound complications, infection, scarring, or recurrence?	Need for hematology input, embolization/sclerotherapy, drug adjustment, or staged surgery	Coagulation status, medication exposure, nutritional status, wound plan [16,18,19]
Reconstruction required	Is the goal excision, debulking, contouring, flap coverage, grafting, scar revision, palliation, or functional restoration?	Defines operative endpoint beyond lesion removal	Operative goal, reconstructive ladder, expected residual disease, revision plan [22-24]
Multidisciplinary follow-up	Who will monitor growth, recurrence, drug toxicity, function, psychosocial health, and transition of care?	Ensures long-term pediatric surveillance after medical or surgical treatment	Team composition, follow-up interval, outcome measures, transition plan [19,20,37,38]

Evidence Gaps and Future Directions

Several gaps limit the translation of targeted therapy into pediatric reconstructive practice. First, drug studies often underreport surgical endpoints such as operative timing, blood loss, wound healing, leakage, staged reconstruction, revision surgery, and whether surgery was avoided or delayed. Second, pediatric growth outcomes are rarely reported systematically, despite their importance in craniofacial, limb, truncal, and genital lesions. Third, perioperative management of sirolimus, alpelisib, and emerging MEK inhibitors remains insufficiently standardized. Fourth, patient- and family-reported outcomes are not consistently integrated into surgical decision-making, even though standardized vascular malformation outcome tools are increasingly available [7,8,38]. Finally, future prospective registries should combine ISSVA classification, genotype, therapy exposure, procedural history, reconstructive endpoints, functional outcomes, and long-term follow-up to determine whether targeted therapy truly changes the child’s lifetime surgical burden [2,3,19,20,37].

Future research should prioritize prospective multicenter registries integrating molecular findings, targeted therapy exposure, surgical interventions, functional outcomes, and long-term pediatric quality-of-life assessment [38-40]. Standardized reporting of operative endpoints, including wound complications, blood loss, revision burden, delayed surgery, and avoided procedures, would help clarify whether targeted therapies truly alter the lifetime reconstructive trajectory of affected children [38,39]. In parallel, advances in molecular diagnostics may support genotype-guided surgical planning, where biologic response patterns influence the timing, extent, and sequencing of debulking and reconstructive procedures [41-47]. Continued collaboration between vascular anomaly specialists, reconstructive surgeons, geneticists, and interventional teams will therefore be essential to establish evidence-based multidisciplinary treatment algorithms in pediatric vascular anomalies [48].

## Conclusions

Targeted therapy has transformed the management of complex pediatric vascular anomalies, but its reconstructive implications remain incompletely defined. Agents such as sirolimus and alpelisib should not be viewed simply as alternatives to surgery. Instead, they may reshape surgical timing, reduce procedural morbidity, facilitate staged reconstruction, delay high-risk operations, avoid selected procedures, or modify the goals of debulking and functional restoration.

For children, success should be measured beyond lesion size. Function, growth, quality of life, recurrence, revision burden, and family-centered outcomes are essential. The proposed PERFORM framework offers a practical approach to integrating targeted therapy response into pediatric surgical decision-making. Future studies should standardize reporting of medical-surgical pathways so that multidisciplinary teams can better determine when surgery should be performed, facilitated, delayed, avoided, or modified.
